# Association of Obstructive Sleep Apnea With White Matter Integrity and Cognitive Performance Over a 4-Year Period in Middle to Late Adulthood

**DOI:** 10.1001/jamanetworkopen.2022.22999

**Published:** 2022-07-20

**Authors:** Min-Hee Lee, Seung Ku Lee, Soriul Kim, Regina E. Y. Kim, Hye Ryeong Nam, Ali T. Siddiquee, Robert J. Thomas, Inha Hwang, Jee-Eun Yoon, Chang-Ho Yun, Chol Shin

**Affiliations:** 1 Institute of Human Genomic Study, College of Medicine, Korea University Ansan Hospital, Ansan, Republic of Korea; 2 Division of Pulmonary, Critical Care and Sleep Medicine, Department of Medicine, Division of Pulmonary, Critical Care & Sleep Medicine, Beth Israel Deaconess Medical Center, Boston, Massachusetts; 3 Department of Neurology, Seoul National University Bundang Hospital and Seoul National University College of Medicine, Seongnam, Republic of Korea; 4 Department of Neurology, Uijeongbu Eulji Medical Center, Uijeongbu, Republic of Korea; 5 Department of Pulmonary Sleep and Critical Care Medicine Disorder Center, College of Medicine, Korea University, Ansan, Republic of Korea

## Abstract

**Importance:**

Obstructive sleep apnea (OSA) is associated with cognitive impairment and brain structural alterations, but longitudinal outcomes are understudied.

**Objective:**

To examine the associations of OSA with cognition and white matter (WM) integrity over a 4-year period.

**Design, Setting, and Participants:**

This prospective cohort study was conducted in a community-based adult population among participants who had both baseline (2011-2014) and 4-year follow-up (2015-2018) polysomnography, diffusion tensor imaging, and cognitive assessment data. Participants with neurological disorders, anomalous findings on brain magnetic resonance imaging, or inadequate quality of the evaluations were excluded. Data were analyzed from March to November 2021.

**Exposures:**

Participants were categorized depending on the presence vs absence of OSA at baseline and follow-up polysomnographic analysis.

**Main Outcomes and Measures:**

The main outcomes were proportional changes over a 4-year period in neuropsychological performance and WM integrity. The neuropsychological assessment battery included verbal and visual memory, verbal fluency, Digit Symbol–coding, Trail Making Test–A, and Stroop Test. WM integrity was assessed by fractional anisotropy, axial, and radial diffusivity. To examine interactions with age and sex, participants were subgrouped by age older than 60 years vs 60 years or younger and men vs women.

**Results:**

A total of 1998 individuals were assessed for eligibility, and 888 were excluded based on exclusion criteria, leaving 1110 participants (mean [SD] age, 58.0 [6.0] years; 517 [46.6%] men) for analysis, including 458 participants grouped as OSA-free, 72 participants with resolved OSA, 163 participants with incident OSA, and 417 participants with persistent OSA. Incident OSA was associated with altered WM integrity and with concomitant changes in sustained attention compared with participants without OSA (eg, change in Digit Symbol–coding test score, –3.2% [95% CI, –5.2% to –1.2%]). Participants with resolved OSA showed better visual recall at the follow-up (change in Visual Reproduction–immediate recall test, 17.5% [95% CI, 8.9% to 26.1%]; change in Visual Reproduction–delayed recall test, 33.1% [95% CI, 11.3% to 54.9%]), with concordant changes in diffusion parameters at the relevant anatomic areas. In the older group only (age >60 years), persistent OSA was associated with altered WM integrity and cognition (eg, Visual Reproduction–recognition test: β = −24.2 [95% CI, −40.7 to −7.7]). Sex also was associated with modifying the association of OSA with WM integrity of the left posterior internal capsule, the left genu of corpus callosum, and the right middle cerebellar peduncle only in men and with cognition only in women (eg, Visual Reproduction–immediate recall test: β = 33.4 [95% CI, 19.1 to 47.7]).

**Conclusions and Relevance:**

These findings suggest that dynamic changes in OSA status were significantly associated with WM integrity and cognition, which varied by age and sex. It is possible that adequate interventions for OSA could better preserve brain health in middle to late adulthood.

## Key Points

**Question:**

Is obstructive sleep apnea (OSA) associated with cognition and white matter (WM) integrity over time?

**Findings:**

In this cohort study of 1110 participants, OSA was associated with impaired cognition and WM integrity during 4 years of follow-up. Incident and persistent OSA were associated with accelerated attention, visual processing, and visual memory decline, which correlated with changes in fractional anisotropy of the relevant WM areas. Age and sex were associated with modifying the associations.

**Meaning:**

These findings suggest that timely evaluation and adequate intervention of OSA could aid in preserving brain health, improving cognition, and reducing the risk of cognitive impairment.

## Teaser

This cohort study assess the associations of obstructive sleep apnea with 4-year changes in cognitive function and white matter integrity among middle-aged and older adults.

## Introduction

Obstructive sleep apnea (OSA) is caused by various driver endotypes^1^ and causes intermittent hypoxia and sleep fragmentation, triggering downstream events, including oxidative stress, systemic inflammation, sympathetic overactivity, dysmetabolism, and hemodynamic swings.^2^ OSA is associated with daytime sleepiness, depression, cognitive impairment, and dementia.^3,4,5,6^ Impaired sleep function underlies cognitive dysfunction in OSA,^6^ from impairment of the precise temporal coordination of slow oscillations, sleep spindles, and hippocampal ripples^7^ and glymphatic flow.^8^ These processes correlate with slow-wave activity and continuity, disrupted by OSA.^9,10,11^ Direct neural injury also contributes to cognitive impairment in OSA,^5,12^ such as reduced gray matter volume or thickness and altered white matter (WM) integrity.^13,14,15,16,17^ However, the findings are inconsistent.^18,19,20,21^ Even regional gray matter hypertrophy has been reported.^22,23,24^ For WM integrity, OSA is generally associated with reduced fractional anisotropy (FA) and increased diffusivity,^16,25,26^ but in some studies, diffusivity was reduced with or without concomitant change in FA values.^27,28,29^ Inconsistent, even opposing, findings could be explained by severity, duration, heterogeneity within OSA and acute transitory response (edema, reactive gliosis, reduced diffusivity) vs chronic neuronal injury (neuronal loss, axonal degeneration, reduced FA, increased diffusivity).^17,30^

We performed overnight polysomnography (PSG), diffusion tensor imaging (DTI), and neuropsychological assessment at 2 time points 4 years apart in a representative sample of the middle-aged and older adult population in Korea to investigate the longitudinal association of OSA with WM and cognition. We hypothesized that the change in cognitive performance and WM integrity differed by OSA status (OSA-free vs resolved, incident, or persistent OSA over 4 years), and that age and sex would be associated with modifying the association.

## Methods

### Study Participants

This cohort study was approved by the institutional review board of Korea University Ansan Hospital. All participants provided written informed consent at the baseline and follow-up visits. This report followed the Strengthening the Reporting of Observational Studies in Epidemiology (STROBE) reporting guideline.

This study is a part of the Korean Genome and Epidemiology Study.^31^ The original cohort was established in Ansan, South Korea, and included 5012 adults aged 40 to 69 years in 2001 to 2002. The participants were evaluated for demographic characteristics, medical history, health status, and sleep-related factors.

### Overnight PSG and OSA Status

All participants underwent PSG at home with portable devices (Embletta X-100; Embla Systems). The methods for PSG scoring were described in detail elsewhere^32^ and in the eAppendix in the Supplement. The presence and severity of OSA were based on the apnea-hypopnea index (AHI), with OSA-free classified as less than 5.0 events/h; mild, 5.0 to 14.9 events/h; moderate, 15.0 to 29.9 events/h; and severe, 30.0 events/h or more.

Changes in OSA status over 4 years were determined by comparing the results of PSG at baseline and follow-up and divided into 4 groups: OSA-free, resolved OSA, incident OSA, and persistent OSA. To examine interactions with age and sex, participants were subgrouped by age older than 60 years vs 60 years or younger at baseline and men vs women. The age cutoff was concordant with the criteria for middle-aged vs older adults in a recent systematic review showing the longitudinal association of OSA with Alzheimer disease.^5^

### Neuropsychological Assessment Battery

The neuropsychological assessment battery consisted of Story Recall (SR), Visual Reproduction (VR), phonemic verbal fluency (VF1) and categorical verbal fluency (VF2), Digit Symbol–coding (DS), Trail Making Test–A (TMA), and Stroop Test–Word Reading (STROOP1) and Stroop Test–Color Reading (STROOP2) tests. The SR and VR tests included immediate recall (IR), delayed recall (DR), and recognition (RECOG). Higher scores indicated better performance, except TMA. The details of cognitive assessment are described elsewhere^33^ and in the eAppendix in the Supplement.

For each cognitive test, we calculated the difference in performance over the 4-year period, (∆*cog*(%) = 100 × (*performance at follow-up* – *performance at baseline*) / *performance at baseline*). For the tests other than TMA, a negative value of *Δcog* represents lower performance and a positive value indicates higher performance at follow-up than baseline.

### FA and Diffusivity Maps

We computed FA, axial diffusivity, and radial diffusivity (RD)^34,35^ and generated maps that illustrated the differences in FA, axial diffusivity, and RD over time (eg, *ΔFA*(%) = 100 × (*FA at follow-up* – *FA at baseline*) / *FA at baseline*) at each voxel. Details on the magnetic resonance imaging (MRI) analysis are in the eAppendix in the Supplement.

### Statistical Analysis

General characteristics were compared between the groups with analysis of variance or χ^2^ test, and longitudinal differences were examined with paired *t* test or χ^2^ (McNemar-Bowker) test.^36^

 Cognitive performance at baseline was compared using analysis of covariance, and the longitudinal association of OSA status with change in cognitive scores was assessed using linear regression. Statistical significance was set at *P* < .05, after false discovery rate was controlled for multiple comparisons. For WM integrity, DTI-derived parameters were compared between the groups voxel-wise. Statistical significance was set at *P* < .05 after Bonferroni correction using a threshold-free cluster enhancement method implemented in FSL with 5000 permutations^37^; the cluster threshold was set at 15 contiguous significant voxels. To understand how each diffusivity contributed to any significant differences in FA (or change in FA) between the groups, the values of axial diffusivity (or change in axial diffusivity) and RD (or change in RD) for the corresponding anatomic locations were provided, regardless of the statistical significance. Covariates adjusted in the multivariable analysis were age, sex, education, body mass index (BMI; calculated as weight in kilograms divided by height in meters squared), current drinking, hypertension, and diabetes.

We conducted partial correlation analyses to investigate the independent association between change in cognitive scores and change in FA of WM regions with significant differences between the groups.

Statistical analyses were conducted using SPSS version 24.0 (SPSS) and MATLAB version R2019b (Mathworks). Data were analyzed from March to November 2021.

## Results

Among 2873 participants who underwent baseline PSG, MRI, and cognitive assessments (2011-2014), 1998 participants completed the follow-up examinations (2015-2018), with a mean (SD) interval of 4.2 (0.5) years. Since the history of neurological or psychiatric disorders and structural brain pathology at baseline may confound the independent association of OSA with cognitive function and WM integrity, we excluded participants who reported a history of neurological or psychiatric disorders (62 participants) and who had significant structural pathological conditions on the baseline MRI, such as a large vessel stroke (11 participants), lacune (62 participants), intracerebral hemorrhage (52 participants), or severe age-related WM change (17 participants). Participants with incomplete cognitive (41 participants) and inadequate PSG (5 participants) assessments were also excluded. Through manual review of DTI quality, we further excluded participants with severe signal loss (38 participants), distortion (418 participants), and motion artifacts (182 participants). Finally, 1110 participants (mean [SD] age, 58.0 [6.0] years at baseline; 517 [46.6%] men) were included (eFigure 1 in the Supplement). The general characteristics at baseline were compared between the included and excluded participants (eTable 1 in the Supplement). The distribution of OSA severity was comparable between the excluded and the included participants, although the AHI was higher in excluded participants (mean [SD] 8.2 [9.5] vs 7.0 [8.5]), and they were older (mean [SD] age, 59.8 [6.9] years) and had more hypertension and diabetes (eTable 1 in the Supplement).

### Changes in the OSA Status

Among 1110 participants, OSA was present in 489 participants (44.0%) at baseline, including 338 participants with mild OSA (69.1%), 125 participants with moderate OSA (25.6%), and 26 participants with severe OSA (5.3%). Among participants with OSA at baseline, only 6 had been treated with continuous positive airway pressure (CPAP) therapy during the study period. A total of 621 participants were free from OSA at baseline. The OSA group, compared with the non-OSA group, was older (mean [SD] age, 59.5 [6.3] years vs 56.9 [5.4] years; *P* < .001), included more men (280 [57.3%] men vs 237 [38.2%] men; *P* < .001), had higher BMI (mean [SD], 25.5 [3.0] vs 24.0 [2.8]; *P* < .001) and AHI (mean [SD], 13.5 [9.3] vs 1.9 [1.4]; *P* < .001), and higher prevalence of hypertension (244 participants [49.9%] vs 194 participants [31.2%]; *P* < .001) and diabetes (173 participants [27.9%] vs 131 participants [21.1%]; *P* < .001). The prevalence of OSA increased to 580 participants (52.3%) over the 4-year period, while OSA status changed in 235 participants (21.2%), resolved in 72 participants (6.5%), and became incident in 163 patients (14.7%) (Table 1).

**Table 1.** Demographic Data and Sleep Variables of the Participants Grouped by the OSA Status

Variable	OSA-free (n = 458), No. (%)^a^		*P* value	Resolved OSA (n = 72), No. (%)^b^		*P* value	Incident OSA (n = 163), No. (%)^c^		*P* value	Persistent OSA (n = 417), No. (%)^d^		*P* value
	Baseline	Follow-up		Baseline	Follow-up		Baseline	Follow-up		Baseline	Follow-up	
Age, mean (SD), y	56.5 (5.1)	60.6 (5.1)	<.001	59.9 (7.1)	64.0 (7.1)	<.001	58.0 (6.2)	62.1 (6.3)	<.001	59.5 (6.2)	63.5 (6.2)	<.001
Sex												
Women	291 (63.5)	NA	NA	31 (43.1)	NA	NA	93 (57.1)	NA	NA	178 (42.7)	NA	NA
Men	167 (36.5)	NA	NA	41 (56.9)	NA	NA	70 (42.9)	NA	NA	239 (57.3)	NA	NA
Education, y												
≤6	41 (9.0)	NA	NA	11 (15.3)	NA	NA	20 (12.3)	NA	NA	47 (11.3)	NA	NA
7-9	96 (21.0)	NA	NA	8 (11.1)	NA	NA	31 (19.0)	NA	NA	73 (17.5)	NA	NA
10-12	225 (49.1)	NA	NA	41 (56.9)	NA	NA	77 (47.2)	NA	NA	198 (47.5)	NA	NA
13-16	84 (18.3)	NA	NA	10 (13.9)	NA	NA	31 (19.0)	NA	NA	86 (20.6)	NA	NA
>16	12 (2.6)	NA	NA	2 (2.8)	NA	NA	4 (2.5)	NA	NA	13 (3.1)	NA	NA
BMI	23.7 (2.6)	23.6 (2.6)	.16	25.2 (2.6)	24.8 (2.6)	.01	24.8 (3.1)	24.8 (3.4)	.99	25.6 (3.0)	25.5 (3.1)	.01
Current smokers	42 (9.2)	38 (8.3)	.42	12 (16.7)	11(15.3)	>.99	18 (11.0)	10 (6.1)	.03	53 (12.7)	38 (9.1)	.005
Current drinkers	173 (37.8)	157 (35.6)	.04	32 (44.4)	25(39.1)	.05	79 (48.5)	70 (45.2)	.15	216 (51.8)	173 (45.3)	<.001
Hypertension	128 (28.0)	157 (34.3)	<.001	34 (47.2)	36(50.0)	.48	66 (40.5)	81 (49.7)	<.001	210 (50.4)	232 (55.6)	<.001
Diabetes	88 (19.2)	106 (23.1)	<.001	31 (43.1)	33(45.8)	.48	43 (26.4)	47 (28.8)	.13	142 (34.1)	167 (40.1)	<.001
ESS												
Mean (SD)	4.9 (3.0)	4.2 (3.2)	<.001	4.8 (3.2)	4.0 (3.3)	.15	5.0 (3.0)	4.6 (3.4)	.28	5.0 (3.1)	4.4 (3.3)	<.001
≥11	21 (4.6)	17 (3.7)	.56	3 (4.2)	1 (1.4)	.62	8 (4.9)	9 (5.5)	>.99	21 (5.0)	23 (5.5)	>.99
BDI												
Mean (SD)	7.2 (6.0)	6.2 (5.9)	<.001	6.4 (6.0)	5.6 (4.9)	.20	8.5 (7.9)	6.1 (6.4)	<.001	7.6 (7.1)	6.1 (6.3)	<.001
Mild (10-16)	98 (21.4)	78 (17.0)	.66	11 (15.3)	10 (13.9)	.80	28 (17.2)	18 (11.0)	.99	78 (18.7)	60 (14.4)	.71
Moderate (17-29)	36 (7.9)	24 (5.2)		5 (6.9)	2 (2.8)		18 (11.0)	11 (6.8)		40 (9.6)	22 (5.3)	
Severe (≥30)	2 (0.4)	1 (0.2)		0 (0.0)	0 (0.0)		2 (1.2)	1 (0.6)		5 (1.2)	5 (1.2)	
AHI												
Mean (SD), events/h	1.6 (1.3)	2.0 (1.4)	<.001	9.3 (4.7)	3.3 (1.2)	<.001	2.7 (1.3)	9.8 (6.3)	<.001	14.3 (9.7)	15.9 (10.5)	<.001
Mild (5-14)	NA	NA	NA	64 (88.9)	NA	NA	NA	143 (87.7)		274 (65.7)	244 (58.5)	.01
Moderate (15-29)	NA	NA	NA	8 (11.1)	NA	NA	NA	16 (9.8)		117 (28.1)	134 (32.1)	
Severe (≥30)	NA	NA	NA	0 (0.0)	NA	NA	NA	4 (2.5)		26 (6.2)	39 (9.4)	
Supine, mean (SD), events/h	3.0 (4.0)	4.0 (4.6)	<.001	18.5 (12.9)	9.8 (9.8)	<.001	5.6 (6.6)	17.4 (13.6)	<.001	24.3 (16.7)	29.0 (17.7)	<.001
Nonsupuine, mean (SD), events/h	0.7 (1.3)	0.9 (1.8)	.06	2.5 (2.7)	1.2 (1.0)	<.001	1.0 (1.0)	3.8 (5.2)	<.001	4.8 (7.0)	6.4 (12.6)	.009
Sao_2_, mean (SD), %												
Overall	96.3 (1.0)	95.8 (1.0)	<.001	95.3 (1.1)	95.4 (1.2)	.73	95.9 (1.3)	95.2 (1.3)	<.001	95.1 (1.2)	94.7 (1.3)	<.001
Minimum	90.3 (5.0)	90.2 (2.8)	.66	85.9 (4.0)	88.4 (2.9)	<.001	88.8 (5.3)	85.9 (3.7)	<.001	83.3 (5.9)	82.8 (4.9)	.04
TST, mean (SD), h	6.4 (1.3)	6.1 (1.3)	<.001	6.3 (1.6)	5.9 (1.4)	.02	6.3 (1.4)	5.8 (1.3)	<.001	6.3 (1.2)	6.0 (1.2)	<.001
Sleep in supine position, mean (SD), %^e^	57.4 (23.6)	52.1 (24.7)	<.001	53.2 (23.5)	38.7 (23.6)	<.001	53.2 (24.7)	56.7 (26.0)	.05	54.1 (23.6)	49.5 (23.6)	<.001

Abbreviations: AHI, apnea-hypopnea index; BDI, Beck Depression Inventory; BMI, body mass index (calculated as weight in kilograms divided by height in meters squared); ESS, Epworth Sleepiness Scale; Sao_2_, oxygen saturation; NA, not applicable; OSA, obstructive sleep apnea; TST, total sleep time.

^a^ Defined as absence of OSA at the baseline and the follow-up study.

^b^ Defined as OSA at the baseline but no OSA at the follow-up.

^c^ Defined as no OSA at the baseline but OSA at the follow-up.

^d^ Defined as presence of OSA at the baseline and the follow-up.

^e^ Calculated as proportion of supine sleep time among total sleep time.

### Factors Associated With OSA Status

Severity of the resolved OSA at baseline and incident OSA at the follow-up was predominantly mild (Table 1). Compared with baseline, supine sleep was lower in the resolved OSA group and higher in the incident OSA at the follow-up (Table 1), but position was not a sole factor associated with AHI differences. In the resolved OSA group, supine AHI decreased from a mean (SD) of 18.5 (12.9) events/h to 9.8 (9.8) events/h, with concurrent reductions in nonsupine AHI (mean [SD], 2.5 [2.7] events/h to 1.2 [1.0] events/h), BMI, and alcohol use (Table 1). In the incident OSA group, whose BMI did not change, AHI increased 3-fold at the follow-up (mean [SD]: supine AHI, 5.6 [6.6] events/h to 17.4 [13.6] events/h; nonsupine AHI, 1.0 [1.0] events/h vs 3.8 [5.2] events/h), with increases in supine sleep.

### Baseline Differences in Cognitive Performance and WM Integrity

Cognitive function did not significantly differ by OSA (eTable 2 in the Supplement), but age and sex were associated with modifying the associations. In the older subgroup (291 participants aged >60 years), OSA was associated with worse VR-RECOG, but in 819 participants aged 60 years or younger, VF2 was better in the OSA group. Men with OSA had lower performance in SR-RECOG and VR-RECOG but excelled in the control group in VF2 and STROOP1 test (eTable 2 in the Supplement). In women, cognitive performance was comparable between OSA and non-OSA groups.

The OSA group had significantly lower FA and higher RD at the bilateral superior corona radiata and bilateral posterior internal capsule (eFigure 2 in the Supplement). Axial diffusivity was lower in the corresponding areas in the OSA group. In the older subgroup, the OSA group had substantially lower FA and axial diffusivity in the right posterior internal capsule and right posterior thalamic radiation, where RD was higher. In the younger subgroup, OSA was associated with significantly lower FA and axial diffusivity and with higher RD in the bilateral posterior internal capsule. In men, FA and axial diffusivity were lower, and RD was higher in the right superior corona radiata in the OSA group. However, women had no differences in FA, axial diffusivity, or RD between OSA and non-OSA groups.

### Differences in Cognition and WM Integrity at 4-Year Follow-up

The OSA-free group was the reference for group comparisons. Changes in cognition at 4 years differed by group: visual memory was better in the resolved OSA group (change in VR–IR test, 17.5% [95% CI, 8.9% to 26.1%]; change in VR-DR test, 33.1% [95% CI, 11.3% to 54.9%]), and accelerated worsening in visual processing and sustained attention on DS tasks were observed in the incident OSA group (eg, change in DS test, –3.2% [95% CI, –5.2% to –1.2%]) (Table 2). Cognitive performances in the persistent OSA remained unchanged.

**Table 2.** Change in Cognitive Performance Over the 4-Year Follow-up Compared by the OSA Status^a^

Domain	Resolved OSA (n = 72)		Incident OSA (n = 163)		Persistent OSA (n = 417)	
	β (95% CI)	*P* value^b^	β (95% CI)	*P* value^b^	β (95% CI)	*P* value^b^
SR-IR	2.0 (–6.6 to 10.6)	.76	0.2 (–6.1 to 6.5)	.94	–1.3 (–6.4 to 3.8)	.84
SR-DR	–5.1 (–13.7 to 3.5)	.50	–5.4 (–11.9 to 1.1)	.37	–2.3 (–7.4 to 2.8)	.65
SR-RECOG	0.8 (–3.5 to 5.1)	.76	–1.4 (–4.5 to 1.7)	.66	–1.7 (–4.3 to 0.9)	.42
VR-IR	17.5 (8.9 to 26.1)	<.001	5.0 (–1.3 to 11.3)	.37	–0.7 (–5.8 to 4.4)	.84
VR-DR	16.1 (5.1 to 27.1)	.02	–2.4 (–10.4 to 5.6)	.66	–4.6 (–11.1 to 1.9)	.42
VR-RECOG	11.6 (–0.4 to 23.6)	.24	–6.1 (–14.9 to 2.7)	.39	0.2 (–6.9 to 7.3)	.96
VF1	0.4 (–6.7 to 7.5)	.91	1.9 (–3.2 to 7.0)	.66	1.0 (–3.1 to 5.1)	.84
VF2	1.6 (–4.1 to 7.3)	.76	–2.7 (–6.8 to 1.4)	.39	–3.2 (–6.5 to 0.1)	.42
DS	–1.1 (–3.8 to 1.6)	.71	–3.2 (–5.2 to –1.2)	.02	–1.2 (–2.8 to 0.4)	.42
TMA	5.9 (–1.2 to 13.0)	.25	1.6 (–3.5 to 6.7)	.66	3.5 (–0.6 to 7.6)	.42
STROOP1	2.4 (–0.3 to 5.1)	.25	–0.3 (–2.3 to 1.7)	.86	–0.3 (–1.9 to 1.3)	.84
STROOP2	0.8 (–1.9 to 3.5)	.76	1.9 (–0.1 to 3.9)	.36	0.7 (–0.9 to 2.3)	.65

Abbreviations: DS, Digit Symbol–coding; OSA, obstructive sleep apnea; DR, delayed recall; IR, immediate recall; RECOG, recognition; SR, Story Recall; STROOP1, Stroop Test–word reading; STROOP2, Stroop Test–color reading; TMA, Trail Making Test–A; VF1, Verbal Fluency–phonemic; VF2, Verbal Fluency–categorical; VR, Visual Reproduction.

^a^ The OSA-free group was used as the reference to assess change.

^b^ Adjusted for age, sex, education, body mass index, current drinking, hypertension, and diabetes. *P* values corrected for false discovery rate.

**Figure 1.**
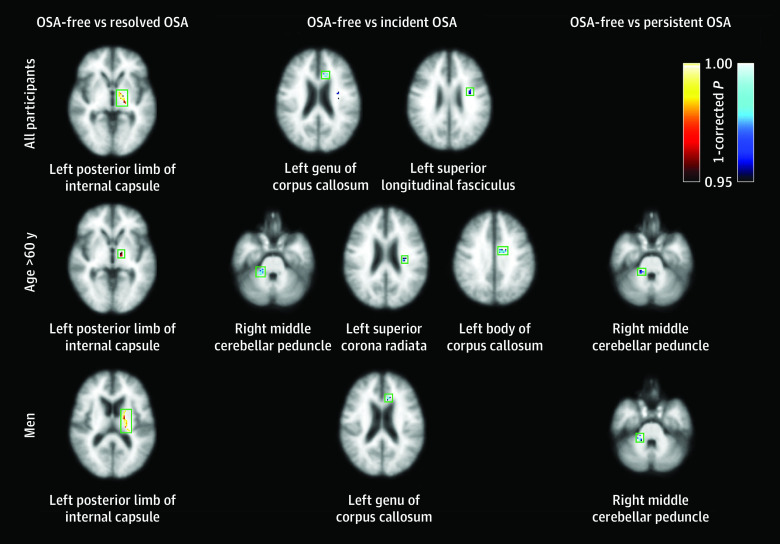
Brain Regions With Significant Changes in Fractional Anisotropy (FA) Over 4 Years in All Participants, in Those Older Than 60 Years, and in Men The degree and direction of changes in FA in the resolved, incident, and persistent obstructive sleep apnea (OSA) groups are compared with the OSA-free group. The color scale indicates the direction of change in FA with statistical significances (*P* < .05 corrected using Bonferroni correction): yellow to red indicates decrease in FA; navy to cyan, increase in FA. Anatomic labels at each axial image designate the location of clusters in green square.

At follow-up in the OSA-free group, global FA was lower by a mean (SD) of 1.2% (12.4%), but global axial diffusivity was higher by a mean (SD) of 2.2% (9.6%) and RD by 2.9% (16.3%) compared with baseline, which is concordant with reported changes in aging.^38^ The 4-year differences of WM integrity in the resolved, incident, and persistent OSA groups are shown in Figure 1 and eTable 3 in the Supplement. In the resolved OSA group, negative changes in FA and axial diffusivity and positive changes in RD were significant at the left posterior internal capsule. The incident OSA group had a significance positive change in FA at the left genu of the corpus callosum and left superior longitudinal fasciculus, wherein the positive change in axial diffusivity and negative change in RD were marginal. There were no regional differences in changes in FA, RD, and axial diffusivity between the persistent OSA and OSA-free groups.

### Age Differences

Age was associated with modifying the associations of OSA with WM integrity (Figure 1 and eTable 3 in the Supplement) and cognition (Table 3). Among the participants aged 60 years or younger, OSA status was not associated with changes in cognitive scores, FA, axial diffusivity, or RD. In the older subgroup, the resolved OSA group had better visual memory and significantly lower FA at the left posterior internal capsule, with lower axial diffusivity and higher RD. Both incident and persistent OSA groups had worse visual recognition (eg, Visual Reproduction–recognition test: incident OSA, β = –33.9 [95% CI, –54.6 to –13.2]; persistent OSA, β = −24.2 [95% CI, −40.7 to −7.7]) and showed significantly higher FA with higher axial diffusivity and lower RD. The areas with significant differences were wider in the incident OSA group (right middle cerebellar peduncle, left superior corona radiata, and left body of corpus callosum) than the persistent OSA group (right middle cerebellar peduncle).

**Table 3.** The Association of OSA With the Changes in Cognitive Performance by Age and Sex Subgroups

OSA group	Domain, change vs OSA-free group												
	SR-IR	SR-DR	SR-RECOG	VR-IR	VR-DR	VR-RECOG	VF1	VF2	DS	TMA	STROOP1	STROOP2
**Age ≤60 y**													
Resolved (n = 41)												
β (95% CI)	–3.6 (–14.2 to 7.0)	–6.2 (–16.6 to 4.2)	–0.2 (–5.3 to 4.9)	2.9 (–6.7 to 12.5)	5.2 (–7.0 to 17.4)	13.4 (–1.1 to 27.9)	1.5 (–7.7 to 10.7)	–3.7 (–11.0 to 3.6)	–2.1 (–5.4 to 1.2)	6.4 (–2.4 to 15.2)	3.1 (–0.4 to 6.6)	–0.5 (–4.0 to 3.0)
*P* value^a^	.74	.57	.93	.74	.69	.53	.86	.64	.57	.57	.53	.86
Incident (n = 120)												
β (95% CI)	–2.5 (–9.4 to 4.4)	–6.5 (–13.4 to 0.4)	–1.9 (–5.2 to 1.4)	5.4 (–0.9 to 11.7)	–2.5 (–10.4 to 5.4)	0.8 (–8.8 to 10.4)	2.9 (–3.0 to 8.8)	–5.0 (–9.5 to –0.5)	–2.6 (–4.8 to –0.4)	2.3 (–3.4 to 8.0)	0.1 (–2.3 to 2.5)	2.5 (0.3 to 4.7)
*P* value^a^	.64	.19	.52	.22	.65	.92	.58	.13	.13	.64	.92	.13
Persistent (n = 271)												
β (95% CI)	–0.1 (–5.8 to 5.6)	–3.0 (–8.5 to 2.5)	–1.4 (–4.1 to 1.3)	0.5 (–4.6 to 5.6)	–5.6 (–12.3 to 1.1)	6.5 (–1.5 to 14.5)	0.6 (–4.3 to 5.5)	–5.1 (–8.8 to –1.4)	–0.5 (–2.3 to 1.3)	5.4 (0.7 to 10.1)	–0.6 (–2.6 to 1.4)	1.8 (0.0 to 3.6)
*P* value^a^	.98	.54	.54	.92	.27	.27	.92	.10	.76	.15	.76	.24
**Age >60 y**													
Resolved (n = 31)												
β (95% CI)	15.8 (–0.9 to 32.5)	6.6 (–11.7 to 24.9)	1.1 (–7.6 to 9.8)	31.1 (11.2 to 51.0)	33.1 (11.3 to 54.9)	–6.7 (–29.7 to 16.3)	–0.3 (–11.7 to 11.1)	11.7 (1.3 to 22.1)	–0.4 (–5.7 to 4.9)	0.7 (–11.9 to 13.3)	1.4 (–3.3 to 6.1)	0.4 (–4.7 to 5.5)
*P* value^a^	.19	.96	.96	.02	.02	.96	.96	.11	.96	.96	.96	.96
Incident (n = 43)												
β (95% CI)	13.4 (–1.8 to 28.6)	1.1 (–16.0 to 18.2)	–0.1 (–8.0 to 7.8)	4.3 (–14.0 to 22.6)	–0.3 (–20.8 to 20.2)	–33.9 (–54.6 to –13.2)	–2.5 (–12.7 to 7.7)	5.8 (–3.5 to 15.1)	–4.9 (–9.8 to 0.0)	–2.2 (–13.8 to 9.4)	–0.7 (–5.0 to 3.6)	–1.5 (–6.2 to 3.2)
*P* value^a^	.34	.98	.98	.98	.98	.02	.98	.65	.34	.98	.98	.98
Persistent (n = 146)												
β (95% CI)	1.7 (–10.1 to 13.5)	4.5 (–8.5 to 17.5)	–2.2 (–8.3 to 3.9)	–6.9 (–20.9 to 7.1)	1.9 (–13.5 to 17.3)	–24.2 (–40.7 to –7.7)	–0.0 (–7.9 to 7.9)	4.7 (–2.6 to 12.0)	–2.2 (–5.9 to 1.5)	–3.9 (–12.8 to 5.0)	0.3 (–3.0 to 3.6)	–2.9 (–6.6 to 0.8)
*P* value^a^	.94	.75	.75	.75	.94	.05	.99	.75	.75	.75	.94	.73
**Men**													
Resolved (n = 41)												
β (95% CI)	0.2 (–12.6 to 13.0)	–2.1 (–13.5 to 9.3)	2.3 (–3.6 to 8.2)	5.1 (–5.1 to 15.3)	17.8 (4.8 to 30.8)	12.0 (–3.9 to 27.9)	–3.7 (–12.9 to 5.5)	3.4 (–4.5 to 11.3)	1.8 (–1.7 to 5.3)	7.5 (–1.9 to 16.9)	3.3 (–0.4 to 7.0)	–0.7 (–4.2 to 2.8)
*P* value^a^	.98	.78	.59	.59	.08	.42	.59	.59	.59	.42	.42	.78
Incident (n = 70)												
β (95% CI)	–1.2 (–11.8 to 9.4)	–5.4 (–15.2 to 4.4)	1.2 (–3.7 to 6.1)	4.3 (–4.1 to 12.7)	–0.5 (–11.3 to 10.3)	–16.5 (–29.5 to –3.5)	–2.7 (–10.4 to 5.0)	–2.1 (–8.6 to 4.4)	–2.9 (–5.8 to 0.0)	–0.3 (–8.2 to 7.6)	0.1 (–3.0 to 3.2)	0.6 (–2.3 to 3.5)
*P* value^a^	.95	.93	.95	.93	.95	.16	.95	.95	.30	.95	.95	.95
Persistent (n = 239)												
β (95% CI)	–3.8 (–11.5 to 3.9)	–3.3 (–10.2 to 3.6)	–0.7 (–4.2 to 2.8)	–0.8 (–6.9 to 5.3)	–1.0 (–8.9 to 6.9)	–5.0 (–14.8 to 4.8)	–0.9 (–6.4 to 4.6)	–4.5 (–9.2 to 0.2)	–0.8 (–3.0 to 1.4)	0.4 (–5.3 to 6.1)	–0.5 (–2.9 to 1.9)	0.6 (–1.6 to 2.8)
*P* value^a^	.88	.88	.88	.88	.88	.88	.88	.74	.88	.89	.88	.88
**Women**													
Resolved (n = 31)												
β (95% CI)	4.0 (–8.4 to 16.4)	–7.3 (–20.7 to 6.1)	–0.5 (–6.8 to 5.8)	33.4 (19.1 to 47.7)	19.8 (1.9 to 37.7)	10.9 (–7.6 to 29.4)	5.4 (–5.8 to 16.6)	–0.9 (–9.7 to 7.9)	–4.8 (–8.9 to –0.7)	1.9 (–8.9 to 12.7)	1.4 (–2.9 to 5.7)	2.8 (–1.7 to 7.3)
*P* value^a^	.71	.58	.88	<.001	.12	.58	.58	.88	.12	.87	.71	.58
Incident (n = 93)												
β (95% CI)	1.3 (–6.6 to 9.2)	–4.9 (–13.5 to 3.7)	–2.8 (–6.7 to 1.1)	5.8 (–3.4 to 15.0)	7.9 (–3.5 to 19.3)	3.6 (–8.4 to 15.6)	4.0 (–2.9 to 10.9)	–1.6 (–7.1 to 3.9)	–3.5 (–6.2 to –0.8)	2.8 (–4.1 to 9.7)	0.1 (–2.6 to 2.8)	2.8 (–0.1 to 5.7)
*P* value^a^	.82	.46	.46	.46	.46	.67	.46	.67	.12	.64	.93	.36
Persistent (n = 178)												
β (95% CI)	2.1 (–5.2 to 9.4)	–1.0 (–8.7 to 6.7)	–1.4 (–4.9 to 2.1)	–1.8 (–10.0 to 6.4)	–6.1 (–16.3 to 4.1)	7.9 (–2.7 to 18.5)	2.8 (–3.3 to 8.9)	–0.3 (–5.2 to 4.6)	–1.7 (–4.1 to 0.7)	5.1 (–1.0 to 11.2)	0.6 (–2.0 to 3.2)	1.6 (–1.0 to 4.2)
*P* value^a^	.80	.88	.76	.80	.58	.58	.73	.90	.58	.58	.80	.58

Abbreviations: DS, Digit Symbol–coding; DR, delayed recall; IR, immediate recall; OSA, obstructive sleep apnea; RECOG, recognition; SR, Story Recall; STROOP1, Stroop Test–word reading; STROOP2, Stroop Test–color reading; TMA, Trail Making Test-A; VF1, Verbal Fluency–phonemic; VF2, Verbal Fluency–categorical; VR, Visual Reproduction.

^a^ Adjusted for age, sex, education, body mass index, current drinking, hypertension, and diabetes. *P* values corrected for false discovery rate.

### Sex Differences

In women, the resolved OSA group had better VR-IR (β = 33.4 [95% CI, 19.1 to 47.7]), but there were no differences in changes in FA, axial diffusivity, or RD by OSA status (Table 3; eTable 3 in the Supplement). In men, changes in cognitive scores in any test were not associated with OSA status at follow-up (Table 3). Men had altered FA, axial diffusivity, and RD at the left posterior internal capsule in the resolved OSA group, at the left genu of corpus callosum in the incident OSA group, and at the right middle cerebellar peduncle in persistent OSA group (eTable 3 in the Supplement).

### Correlations of Longitudinal Differences in Cognitive Performance With WM Integrity

**Figure 2.**
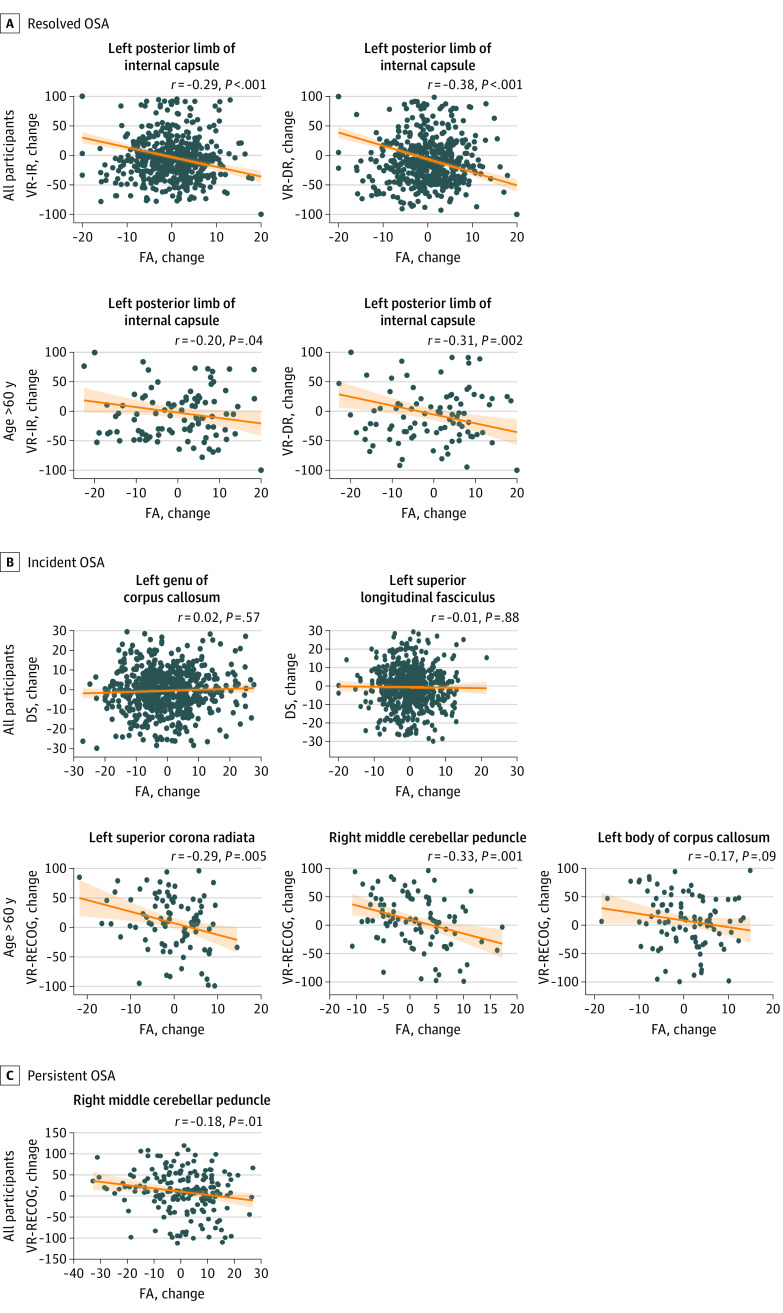
Associations of 4-Year Changes in Cognitive Performance With Change in Fractional Anisotropy (FA) The changes in visual memory correlated with changes in FAs at most of the anatomic areas associated with obstructive sleep apnea (OSA) status. DR indicates delayed recall; DS, Digit Symbol–coding; IR, immediate recall; RECOG, recognition; VR, Visual Reproduction.

Change in FA at the left posterior limb of internal capsule significantly correlated with changes in VR-IR (*r* = –0.29; *P* < .001) and VR-DR (*r* = –0.38; *P* < .001) in the resolved OSA group (Figure 2). The incident OSA group showed no correlation between changes in DS and FA. For the older subgroup, changes in FA in the left posterior limb of internal capsule significantly correlated with changes in VR-IR and VR-DR in the resolved OSA group. In the incident OSA group, change in VR-RECOG correlated with changes in FA in the right middle cerebellar peduncle, left superior corona radiata, and left body of corpus callosum.

## Discussion

In this cohort study assessing a representative sample of middle-aged or older adults in the general Korean population over 4 years,^39^ resolved and incident OSA were associated with altered WM integrity and cognition. An association of persistent OSA with cognition and cerebral WM integrity was evident in the older subgroup (>60 years). Sex was associated with modifying the associations of WM integrity in men and cognitive function in women. Regional DTI metrics correlated with the cognitive performances in the relevant domains.

At baseline, OSA was associated with reduced FA and increased RD and axial diffusivity. Dismantled myelin, loosely packed axonal fibers, and widened interstitial space facilitates water diffusion perpendicular to, as well as in the direction of, axonal fibers, but fragmented axons or reduced axonal density might limit axial diffusion,^17,29,40^ which could explain longitudinal differences in RD and axial diffusivity, resulting in lower FA. Considering this finding and the suggested pathophysiological processes,^40,41^ it can be speculated that accumulated exposure to OSA exacerbates disruption of WM integrity, further reducing FA.^17^ In this cohort study, 4-year differences in the DTI-derived metrics were significantly associated with OSA status, but their directions were intriguing. In the resolved OSA group, reversal of the WM derangement was expected—higher FA along with lower axial diffusivity and RD at follow-up.^14,28^ The observed findings were opposite to the predicted differences: lower FA and higher RD with a lower axial diffusivity at the follow-up. The differences in the incident OSA group also deviated from the expected course, showing significantly higher FA with higher axial diffusivity and lower RD compared with the OSA-free group at the follow-up. In the persistent OSA group, the degree and the direction of differences in DTI metrics were comparable with the OSA-free group.

The observed differences in the diffusion property could be potentially explained by biphasic acute vs chronic and mixed nature of pathologic processes imposed by OSA.^17,30^ For resolved OSA, withdrawal of acute transitory responses might restore RD previously restricted by gliosis and cytotoxic edema and reverse axial diffusivity previously enhanced by intracellular edema, leading to lower FA compared with the OSA-free group at follow-up.

Age modifications and phasic and mixed responses could potentially explain the DTI profile in the persistent OSA group. A disproportionate age distribution might explain the null findings of no differences between the persistent OSA and OSA-free groups. In the older subgroup, persistent OSA was associated with higher FA with higher axial diffusivity and lower RD, indicating an acute reactive response to OSA in the right cerebellar peduncle, which was one of the structures showing higher FA in the incident OSA. Considering the severity of OSA in the incident and persistent OSA groups, the short-term response could be more profound in persistent OSA, having wider areas with altered WM integrity compared with incident OSA. Prior studies have noted mixed pathological responses in OSA: hypoxia-related cortical thinning and increases in gray matter volume associated with sleep fragmentation or sympathetic activity.^42,43^ Hypoxia and sleep fragmentation can lead to both hypotrophy and hypertrophy but in different anatomic areas.^23^ DTI studies in people with OSA should be carefully interpreted in the context of chronicity, age, sex, and severity, with a mix of relatively acute, compensatory, and finally degenerative changes.^16,25,26,27,28,29,44,45^

Even a mild degree of OSA was associated with substantial changes in brain structure and function, yet there were no differences in diffusion properties or cognitive performance in participants with persistent OSA who had moderate severity. A difference in diffusivity may be more prominent in mild OSA than moderate to severe OSA, and a decrease in extracellular free water, suggesting that cytotoxic edema may be greater in people with mild OSA.^29^

The associations of OSA with cognition were largely consistent in middle-aged adults but variable in older adults, as the associations may be modified by individual characteristics (eg, OSA severity, comorbidity, clinical vs general population).^5^ As OSA prevalence increases with age, the older subgroup may have recently developed or progressed OSA, showing acute transitory process on the DTI and failing to adapt for the OSA-related hypoxemia and sleep disruption.^17,46,47^ For the modification of sex on associations, previous studies have largely recruited participants from sleep clinics, which predominantly treat men because of the high prevalence and correlated symptoms of OSA in men.^25,26,27,28,29,44,45^ In a 2012 report by Macey et al,^44^ moderate OSA was associated with reduced FA in women but not in men. Sex-specific lateralization has been reported for the gray matter hypertrophy or hypotrophy in hippocampal subregions.^48^ Frontal thinning is found only in women with OSA.^49^

### Limitations

This study has several limitations. Our participants were not a clinical population, had mainly mild OSA, and were not particularly sleepy at the time of testing; therefore, our results do not readily generalize to the symptomatic clinical or severe OSA populations. A retest effect is pausible,^50^ although 4 years is a long time for such an effect. OSA severity can be impacted by night-to-night variability and sleep position preferences over time, but the direction of changes in cognitive and DTI-derived metrics in the resolved and incident OSA group excludes a systematic bias laden only by OSA misclassification. The sample sizes of the resolved and incident OSA groups were relatively small. Because of the large proportion of excluded study participants, we cannot completely rule out possible selection bias. It is difficult to determine the direction of association between OSA and WM integrity in this study. As insurance coverage in Korea for sleep apnea clinical treatment started only in 2018, only 6 participants had been treated with CPAP during the study period, and potentials associations of CPAP use with cognition and WM integrity could not be estimated.

## Conclusions

This cohort study found that OSA was associated with differences in cognitive performance and WM integrity over time, especially in the older subgroup. These findings could provide potential targets for intervention to preserve brain health.

## Notes

Supplement. **eAppendix.** Supplementary Methods
eReferences.
**eFigure 1.** Flowchart of Recruitment and Progress of Cohort Participants **eFigure 2.** Brain Regions With Factional Anisotropy (FA) Values Significantly Lower in Obstructive Sleep Apnea (OSA) at Baseline in All Participants, in Age Subgroups, and in Men **eTable 1.** Differences in Demographic and Sleep Characteristics Between Included and Excluded Participants **eTable 2.** Differences in Cognitive Performance Between the non-OSA and the OSA Groups in All Participants and in Subgroups by Age and Sex at Baseline **eTable 3.** Associations of OSA With Longitudinal Change in Fractional Anisotropy (ΔFA), Axial Diffusivity (ΔAD), and Radial Diffusivity (ΔRD)
