# Supplementary material for: Association of Obstructive Sleep Apnea With White Matter Integrity and Cognitive Performance Over a 4-Year Period in Middle to Late Adulthood
Source: JAMA Netw Open. 2022 Jul 20;5(7):e2222999. doi: 10.1001/jamanetworkopen.2022.22999 (PMC9301517; doi:10.1001/jamanetworkopen.2022.22999)
Supplement: Supplement. — eAppendix. Supplementary Methods eReferences. eFigure 1. Flowchart of Recruitment and Progress of Cohort Participants eFigure 2. Brain Regions With Factional Anisotropy (FA) Values Significantly Lower in Obstructive Sleep Apnea (OSA) at Baseline in All Participants, in Age Subgroups, and in Men eTable 1. Differences in Demographic and Sleep Characteristics Between Included and Excluded Participants eTable 2. Differences in Cognitive Performance Between the non-OSA and the OSA Groups in All Participants and in Subgroups by Age and Sex at Baseline eTable 3. Associations of OSA With Longitudinal Change in Fractional Anisotropy (ΔFA), Axial Diffusivity (ΔAD), and Radial Diffusivity (ΔRD) [file jamanetwopen-e2222999-s001.pdf]

Supplemental Online Content

Lee MH, Lee SK, Kim S, et al. Association of obstructive sleep apnea with white matter integrity and cognitive performance over a 4-year period in middle to late adulthood. *JAMA Netw Open*. 2022;5(7):e2222999. doi:10.1001/jamanetworkopen.2022.22999

eAppendix. Supplementary Methods

eReferences.

eFigure 1. Flowchart of Recruitment and Progress of Cohort Participants

eFigure 2. Brain Regions With Factional Anisotropy (FA) Values Significantly Lower in Obstructive Sleep Apnea (OSA) at Baseline in All Participants, in Age Subgroups, and in Men

eTable 1. Differences in Demographic and Sleep Characteristics Between Included and Excluded Participants

eTable 2. Differences in Cognitive Performance Between the non-OSA and the OSA Groups in All Participants and in Subgroups by Age and Sex at Baseline

eTable 3. Associations of OSA With Longitudinal Change in Fractional Anisotropy (ΔFA), Axial Diffusivity (ΔAD), and Radial Diffusivity (ΔRD)

This supplemental material has been provided by the authors to give readers additional information about their work.

© 2022 Lee MH et al. *JAMA Network Open.*

eAppendix. Supplementary Methods

Methods

Defining apnea and hypopnea on polysomnography (PSG)

Apnea was defined when airflow reduced by ≥ 90% of the baseline for at least 10 secs with ongoing respiratory efforts and hypopneas were scored when reduction of airflow at the minimum 30% for ≥ 10 sec was associated with ≥ 4% desaturation. Apnea-hypopnea index (AHI) was calculated by averaging the number of obstructive apneas and hypopneas per hour of sleep.

Neuropsychological assessment battery

Verbal memory was evaluated with Story Recall (SR) tests that has been standardized and recognized to be analogous to the Wechsler memory scale-third edition.[e1] Verbal Fluency assessment consisted of phonemic (VF1) and categorical fluency (VF2) tests from the Controlled Oral Word Association Test.[e2] Visual memory was measured with the Visual Reproduction (VR) test from the Wechsler memory scale-revised. Visual processing and sustained attention were evaluated by Digit Symbol-coding (DS) test performed according to the Wechsler Adult Intelligence Scale-fourth edition protocol. Simple attention was measured with the Trail Making Test-A (TMA; number sequencing) and Stroop Test-Word Reading (STROOP1). Executive function was assessed with the Stroop Test-Color reading (STROOP2).[e2-e4]

Magnetic resonance imaging (MRI) data acquisition and processing

Magnetic resonance imaging (MRI) scanning was performed with a 1.5-Tesla scanner (General Electric, Milwaukee, WI). The interval between MRI and PSG was 8.0±16.4 days, and the cognitive assessment was performed 15.1±34.0 days after MRI. T1-weighted images were

© 2022 Lee MH et al. *JAMA Network Open.*

acquired with the following parameters: TR=7.7ms, TE=3.4ms, flip angle=12°, slice thickness=1.6mm. Diffusion tensor imaging (DTI) data were achieved at TR=15000ms, TE=93.8ms, 15 isotropic gradient directions with b=1000s/mm^2, and single b=0 image acquisition. DTI artifacts including B1 field inhomogeneity, motion artifacts, susceptibility- and eddy current-induced distortions in DTI data were corrected by using the FSL tool (https://fsl.fmrib.ox.ac.uk/fsl/fslwiki/).[e5-e7]

Fractional anisotropy and diffusivity maps

Fractional anisotropy (FA) measures directionality of water movement in brain tissues, indicating the microstructural integrity of brain tissues since it is sensitive to fiber number, axonal diameter, and myelination.[e8-e10] Axial diffusivity (AD) and radial diffusivity (RD) assesses diffusivity in the direction parallel or perpendicular to the fiber tract, respectively. AD reflects axonal integrity and RD represents myelin integrity, both affected by intracellular/extracellular edema and changes in cellular density and array (neuronal loss, reactive gliosis).[e9,e11]

After creation, the FA maps were registered to the Montreal Neurological Institute space using symmetric diffeomorphic image registration algorithm provided through the Advanced Normalization Tools package.[e12] T1-weighted images of each subject were registered to the individual b0 images and then the individually registered images were transformed to the template space. The resulting registration parameters were applied to corresponding maps. The registered maps were smoothed (Gaussian filter, full-width half maximum=5mm) to minimize registration errors. A population-based white matter (WM) mask image which met FA>0.2 threshold across all subjects was created.

© 2022 Lee MH et al. *JAMA Network Open.*

General characteristics and covariates

Body mass index (BMI, kg/m^2) was calculated from height (cm) and weight (kg) measured in the morning after overnight fasting. The level of education was categorized (≤6, 7–9, 10–12, 13–16, >16 education-year). Smoking and alcohol drinking status were dichotomized (current vs. never or former). Depressive mood was evaluated with the Beck Depression Inventory.[e13] Daytime sleepiness was assessed using the Epworth Sleepiness Scale (ESS).[e14] Scores of over 10 indicated excessive daytime sleepiness. Hypertension was diagnosed when systolic or diastolic blood pressure was equal to or above 140 or 90 mmHg, respectively, or participants took antihypertensive medications. Diabetes mellitus was defined as using oral hypoglycemic agents or insulin, or with fasting blood glucose equal to or above 126 mg dL.

© 2022 Lee MH et al. *JAMA Network Open.*

eReferences

e1. An H, Chey J. A standardization study of the story recall test in the elderly Korean population. *Korean J Clin Psychol* 2004;23:435-454.

e2. Lee JH, Lee KU, Lee DY, et al. Development of the Korean version of the consortium to establish a registry for Alzheimer's disease assessment packet (CERAD-K): Clinical and neuropsychological assessment batteries. *J Gerontol B Psychol Sci Soc Sci* 2002;57:P47-P53.

e3. Seo EH, Lee DY, Kim KW, et al. A normative study of the trail making test in Korean elders. *Int J Geriatr Psychiatry* 2006;21:844-852.

e4. Seo EH, Lee DY, Choo IH, et al. Normative study of the stroop color and word test in an educationally diverse elderly population. *Int J Geriatr Psychiatry* 2008;23:1020-1027.

e5. Andersson JLR, Graham MS, Drobnjak I, Zhang H, Filippini N, Bastiani M. Towards a comprehensive framework for movement and distortion correction of diffusion MR images: within volume movement. *Neuroimage* 2017;152:450-466.

e6. Andersson JLR, Graham MS, Zsoldos E, Sotiropoulos SN. Incorporating outlier detection and replacement into a non-parametric framework for movement and distortion correction of diffusion MR images. *Neuroimage* 2016;141:556-572.

e7. Andersson JLR, Sotiropoulos SN. An integrated approach to correction for off-resonance effects and subject movement in diffusion MR imaging. *Neuroimage* 2016;125:1063-1078.

e8. Le Bihan D, Mangin JF, Poupon C, et al. Diffusion tensor imaging: Concepts and applications. *J Magn Reson Imaging* 2001;13:534-546.

e9. Song SK, Sun SW, Ju WK, Lin SJ, Cross AH, Neufeld AH. Diffusion tensor imaging detects and differentiates axon and myelin degeneration in mouse optic nerve after retinal ischemia. *Neuroimage* 2003;20:1714-1722.

© 2022 Lee MH et al. *JAMA Network Open.*

e10. Song SK, Sun SW, Ramsbottom MJ, Chang C, Russell J, Cross AH. Dysmyelination revealed through MRI as increased radial (but unchanged axial) diffusion of water. *Neuroimage* 2002;17:1429-1436.

e11. Baril AA, Gagnon K, Descoteaux M, et al. Cerebral white matter diffusion properties and free-water with obstructive sleep apnea severity in older adults. *Hum Brain Mapp* 2020;41:2686-2701.

e12. Avants B, Gee JC. Geodesic estimation for large deformation anatomical shape averaging and interpolation. *Neuroimage* 2004;23(Suppl 1):S139-S150.

e13. Beck AT, Steer RA. Internal consistencies of the original and revised beck depression inventory. *J Clin Psychol* 1984;40:1365-1367.

e14. Johns MW. A new method for measuring daytime sleepiness: the Epworth sleepiness scale. *Sleep* 1991;14:540-545.

© 2022 Lee MH et al. *JAMA Network Open.*

eFigure 1. Flowchart of Recruitment and Progress of Cohort Participants

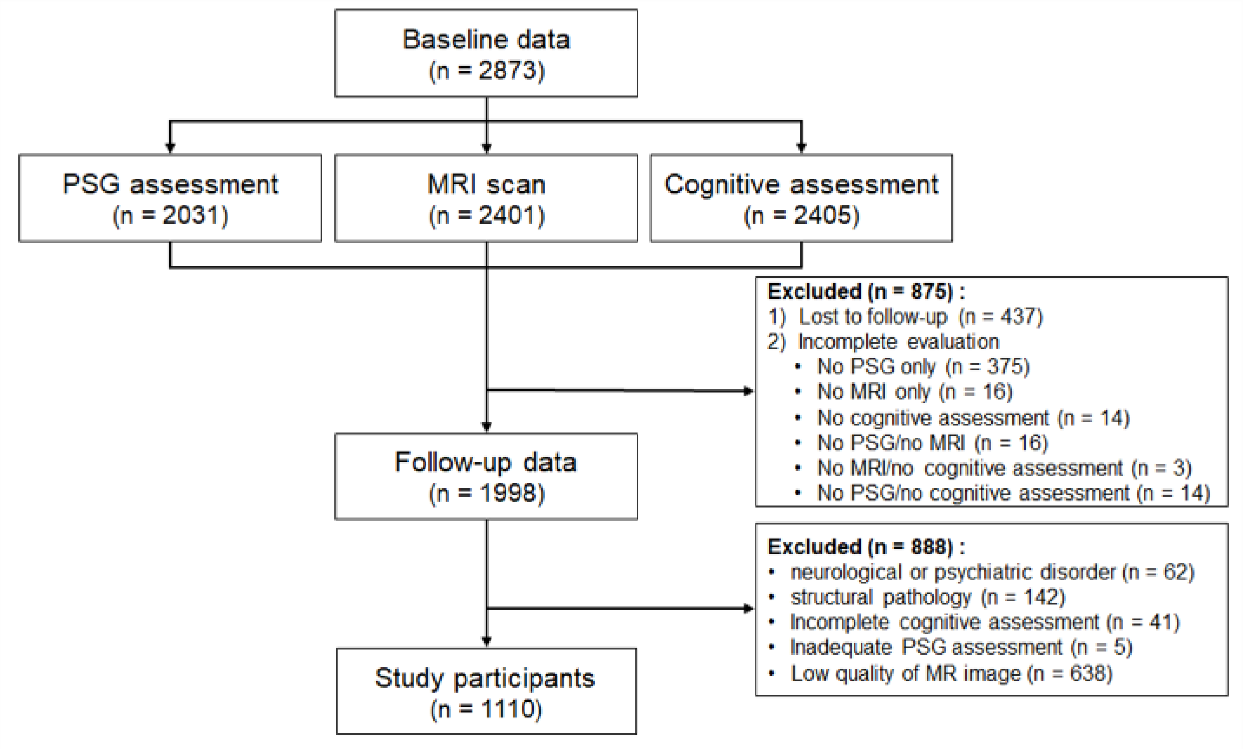

Flow diagram for recruitment, loss to follow-up among cohort participants. Reasons for ineligibility included neurological or psychiatric disorder (n=62), structural pathology (n=142), incomplete cognitive assessment (n=41), inadequate polysomnography (PSG) assessment (n=5), and low quality of diffusion parameters of the magnetic resonance (MR) image (n=638).

© 2022 Lee MH et al. *JAMA Network Open.*

eFigure 2. Brain Regions With Factional Anisotropy (FA) Values Significantly Lower in Obstructive Sleep Apnea (OSA) at Baseline in All Participants, in Age Subgroups, and in Men

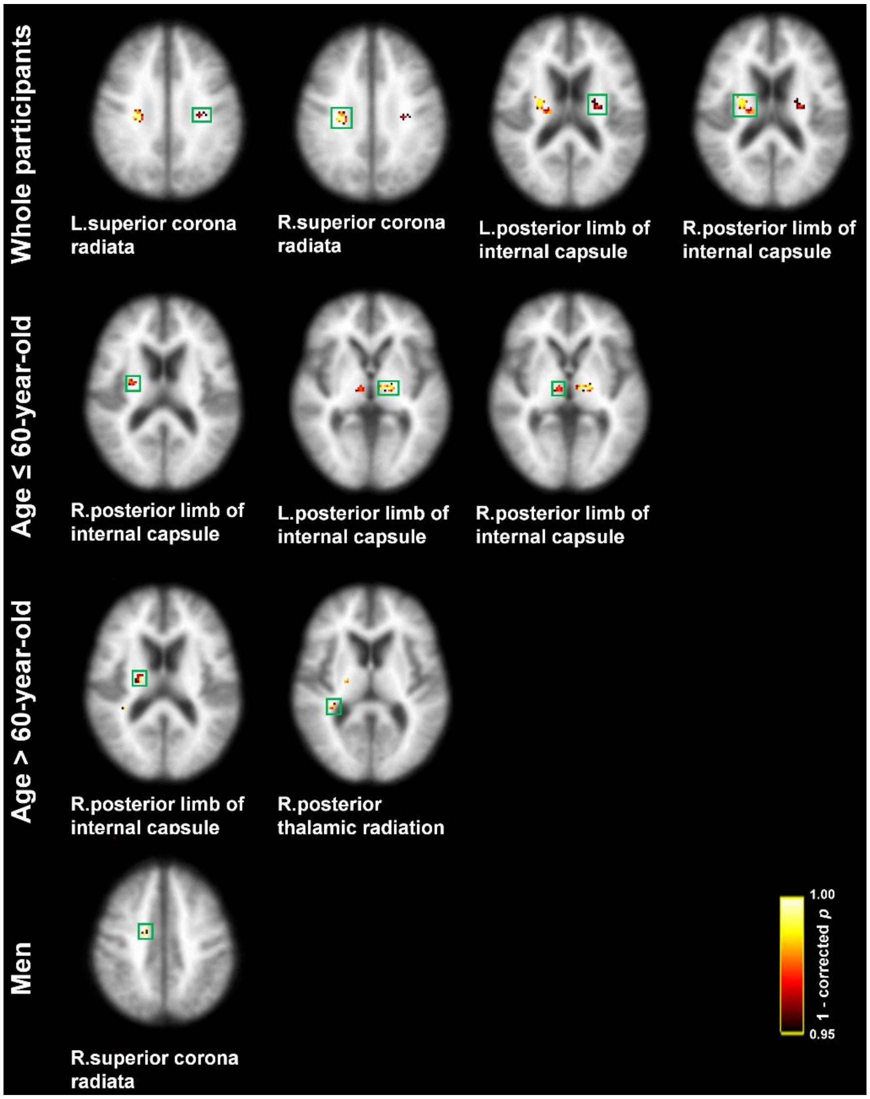

No significant difference in the FA values between OSA and non-OSA in women (not shown). Anatomic label at each axial image designates the location of clusters in the green square. The color-spectrum bar in the right lower corner indicates the level of statistical significance corrected using Bonferroni correction, the yellow-red color being a significantly lower FA in the OSA group (corrected $p<0.05$). *Definition of abbreviations*: R = right; L = left

© 2022 Lee MH et al. *JAMA Network Open.*

eTable 1. Differences in Demographic and Sleep Characteristics Between Included and Excluded Participants

	Included participants (n=1,110)	Excluded participants (n=888)	*p*
Age (years)	58.0±6.0	59.8±6.9	<0.01
Women/Men	593 (53.4) / 517 (46.6)	444 (50.0) / 444 (50.0)	0.13
Education (years)	-	-	-
≤6	119 (10.7)	127 (14.3)	0.02
7-9	208 (18.7)	192 (21.6)	
10-12	541 (48.7)	386 (43.5)	
13-16	211 (19.0)	167 (18.8)	
>16	31 (2.8)	16 (1.8)	
BMI	24.7±3.0	24.7±3.0	0.80
Current smokers	125 (11.3)	101 (11.4)	0.94
Current drinkers	500 (45.1)	412 (46.4)	0.55
Hypertension	438 (39.5)	444 (50.0)	<0.01
Diabetes mellitus	304 (27.4)	299 (33.7)	<0.01
AHI (events/hour)	7.0±8.5	8.2±9.5	<0.01
Mild (5-14)	338 (30.5)	295 (33.2)	0.22
Moderate (15-29)	125 (11.3)	111 (12.5)	
Severe (≥30)	26 (2.3)	36 (4.1)	
$_{min}SaO_2$ (%)	87.2±6.2	86.9±6.0	0.33
TST (hour)	6.4±1.3	6.3±1.2	0.78

Data are presented as n (%) or mean±SD.

Definition of abbreviations: BMI = body mass index; AHI = apnea-hypopnea index; $_{min}SaO_2$ = minimum oxygen saturation; TST = total sleep time

© 2022 Lee MH et al. *JAMA Network Open.*

eTable 2. Differences in Cognitive Performance Between the non-OSA and the OSA Groups in All Participants and in Subgroups by Age and Sex at Baseline

Whole participants	Non-OSA (n = 621)			OSA (n = 489)		p^a
SR-IR	12.2±4.8			12.0±4.7		0.57
SR-DR	11.7±4.8			11.5±4.8		0.68
SR-RECOG	8.3±1.3			8.1±1.4		0.93
VR-IR	7.9±3.0			7.8±3.0		0.30
VR-DR	6.9±3.2			6.8±3.2		0.33
VR-RECOG	2.6±1.1			2.5±1.1		0.17
VF1	26.6±10.2			26.3±9.3		0.57
VF2	15.2±3.7			15.3±3.9		0.06
DS	61.3±17.2			58.4±17.1		0.40
TMA	34.1±12.8			35.3±13.0		0.11
STROOP1	109.2±20.6			107.2±19.6		0.22
STROOP2	52.8±11.6			49.9±11.8		0.69
Subgroup by age	Age ≤ 60			Age > 60		
	Non-OSA (n =507)	OSA (n =312)	p^a	Non-OSA (n = 114)	OSA (n = 177)	p^a
SR-IR	12.3±4.8	12.7±4.7	0.19	11.4±4.6	10.8±4.3	0.21
SR-DR	11.8±4.8	12.2±4.9	0.22	11.0±4.8	10.2±4.5	0.14
SR-RECOG	8.3±1.3	8.3±1.4	0.84	8.0±1.3	7.9±1.4	0.71
VR-IR	8.1±2.9	8.4±2.7	0.38	6.7±3.0	6.8±3.2	0.82
VR-DR	7.1±3.1	7.5±3.0	0.31	5.8±3.4	5.8±3.4	0.86
VR-RECOG	2.7±1.1	2.7±1.1	0.25	2.2±0.9	2.1±1.1	0.02
VF1	26.8±10.4	27.5±9.6	0.16	24.8±8.5	24.0±8.2	0.45
VF2	15.2±3.7	15.8±3.9	0.01	14.3±2.5	14.6±3.6	0.57
DS	64.4±16.1	64.6±15.5	0.22	47.3±14.8	47.4±13.9	0.88
TMA	32.1±10.6	31.1±10.2	0.06	41.3±13.3	41.2±11.3	0.88
STROOP1	111.1±20.0	111.5±18.1	0.11	101.7±18.4	101.7±16.6	0.80
STROOP2	54.2±11.3	52.4±11.7	0.85	46.6±10.3	45.6±9.9	0.53
Subgroup by sex	Men			Women		
	Non-OSA (n =237)	OSA (n =280)	p^a	Non-OSA (n = 384)	OSA (n = 209)	p^a
SR-IR	12.0±4.8	12.2±4.7	0.64	12.3±4.7	11.8±4.6	0.72
SR-DR	11.9±4.8	11.7±4.9	0.87	11.6±4.8	11.1±4.7	0.38
SR-RECOG	8.6±1.0	8.3±1.3	0.01	8.4±1.2	8.1±1.4	0.82
VR-IR	8.4±2.9	8.3±2.8	0.99	7.5±3.0	7.2±3.2	0.20
VR-DR	7.5±3.0	7.2±3.1	0.48	6.5±3.2	6.3±3.3	0.07
VR-RECOG	2.7±1.1	2.6±1.2	0.05	2.7±0.9	2.5±1.0	0.58
VF1	26.5±9.6	27.7±9.1	0.09	25.7±9.2	23.6±8.0	0.39
VF2	15.1±3.7	15.6±3.7	0.04	15.1±3.4	14.6±3.6	0.83
DS	61.5±15.5	62.0±16.8	0.22	61.0±17.7	52.9±15.3	0.64
TMA	32.1±9.1	31.8±8.9	0.16	32.9±9.9	36.4±11.6	0.92
STROOP1	108.7±18.4	110.8±16.2	0.03	109.6±19.5	105.3±16.3	0.84
STROOP2	50.7±10.5	50.6±11.5	0.37	53.9±11.4	49.2±10.3	0.16

Data are presented as mean±SD. Higher scores indicate better performance except TMA. [a]Adjusted for age, sex, education, BMI, current drinking, hypertension, and diabetes.

Definition of abbreviations: SR-IR = Story recall-immediate recall; SR-DR = Story recall-delayed recall; SR-RECOG = Story recall-recognition; VR-IR = Visual reproduction-immediate recall; VR-DR = Visual reproduction-delayed recall; VR-RECOG = Visual reproduction-recognition; VF1 = Verbal fluency-phonemic; VF2 = Verbal fluency-categorical; DS = Digit symbol-coding; TMA = Trail making test-A; STROOP1 = Stroop test-word reading; STROOP2 = Stroop test-color reading

© 2022 Lee MH et al. *JAMA Network Open.*

eTable 3. Associations of OSA With Longitudinal Change in Fractional Anisotropy (ΔFA), Axial Diffusivity (ΔAD), and Radial Diffusivity (ΔRD)

	Resolved OSA		Incident OSA		Persistent OSA	
Whole participants	Region	Δ (Ref.)	Region	Δ (Ref.)	Region	Δ (Ref.)
ΔFA	L.posterior limb of internal capsule[a]	-6.0 (-2.9)	L.genu of corpus callosum[a]	4.6 (-2.9)	NS.	
			L.superior longitudinal fasciculus[a]	3.7 (-1.2)		
ΔAD	L.posterior limb of internal capsule[a]	-1.8 (0.2)	L.genu of corpus callosum[b]	5.5 (4.2)		
			L.superior longitudinal fasciculus[b]	3.2 (2.6)		
ΔRD	L.posterior limb of internal capsule[a]	6.4 (3.8)	L.genu of corpus callosum[b]	-0.3 (1.9)		
			L.superior longitudinal fasciculus[b]	-0.4 (0.7)		
Age>60-year-old	Region	Δ (Ref.)	Region	Δ (Ref.)	Region	Δ (Ref.)
ΔFA	L.posterior limb of internal capsule[a]	-8.4 (-1.6)	R.middle cerebellar peduncle[a]	9.3 (-0.3)	R.middle cerebellar peduncle[a]	5.9 (-4.1)
			L.superior corona radiata[a]	5.3 (0.5)		
			L.body of corpus callosum[a]	3.8 (-2.5)		
ΔAD	L.posterior limb of internal capsule[b]	-2.2 (-0.2)	R.middle cerebellar peduncle[b]	5.5 (2.3)	R.middle cerebellar peduncle[b]	4.2 (2.2)
			L.superior corona radiata[b]	4.2 (1.7)		
			L.body of corpus callosum[b]	4.5 (1.0)		
ΔRD	L.posterior limb of internal capsule[b]	7.1 (2.3)	R.middle cerebellar peduncle[b]	-2.6 (2.0)	R.middle cerebellar peduncle[b]	-1.0 (5.2)
			L.superior corona radiata[b]	-2.7 (-0.2)		
			L.body of corpus callosum[b]	-0.7 (3.3)		
Age≤60-year-old	Region	Δ (Ref.)	Region	Δ (Ref.)	Region	Δ (Ref.)
ΔFA	NS.		NS.		NS.	
ΔAD						
ΔRD						
Men	Region	Δ (Ref.)	Region	Δ (Ref.)	Region	Δ (Ref.)
ΔFA	L.posterior limb of internal capsule[a]	-6.4 (-3.7)	L.genu of corpus callosum[a]	7.1 (-1.4)	R.middle cerebellar peduncle[a]	6.0 (-2.1)
ΔAD	L.posterior limb of internal capsule[a]	-1.8 (1.0)	L.genu of corpus callosum[b]	5.5 (4.1)	R.middle cerebellar peduncle[b]	3.3 (1.9)
ΔRD	L.posterior limb of internal capsule[a]	7.8 (3.9)	L.genu of corpus callosum[b]	-1.6 (2.2)	R.middle cerebellar peduncle[b]	-1.1 (3.2)
Women	Region	Δ (Ref.)	Region	Δ (Ref.)	Region	Δ (Ref.)
ΔFA	NS.		NS.		NS.	
ΔAD						
ΔRD						

Data are presented as mean (%). Different superscript symbols indicate statistical significance at different levels: p-corrected for false discovery rate ≤0.05[a] and uncorrected p≤0.05[b].

Definition of abbreviations: OSA = obstructive sleep apnea; Ref. = the reference value from the OSA-free group; NS. = non-significant L = left; R = right

© 2022 Lee MH et al. *JAMA Network Open.*
